# Endothelium as a Potential Target for Treatment of Abdominal Aortic Aneurysm

**DOI:** 10.1155/2018/6306542

**Published:** 2018-04-03

**Authors:** Jingyuan Sun, Hongping Deng, Zhen Zhou, Xiaoxing Xiong, Ling Gao

**Affiliations:** ^1^Endocrinology & Metabolism Department, Renmin Hospital of Wuhan University, Wuhan, China; ^2^Vascular Surgery Department, Renmin Hospital of Wuhan University, Wuhan, China; ^3^Neurosurgery Department, Renmin Hospital of Wuhan University, Wuhan, China

## Abstract

Abdominal aortic aneurysm (AAA) was previously ascribed to weaken defective medial arterial/adventitial layers, for example, smooth muscle/fibroblast cells. Therefore, besides surgical repair, medications targeting the medial layer to strengthen the aortic wall are the most feasible treatment strategy for AAA. However, so far, it is unclear whether such drugs have any beneficial effect on AAA prognosis, rate of aneurysm growth, rupture, or survival. Notably, clinical studies have shown that AAA is highly associated with endothelial dysfunction in the aged population. Additionally, animal models of endothelial dysfunction and endothelial nitric oxide synthase (eNOS) uncoupling had a very high rate of AAA formation, indicating there is crucial involvement of the endothelium and a possible pharmacological solution targeting the endothelium in AAA treatment. Endothelial cells have been found to trigger vascular wall remodeling by releasing proteases, or recruiting macrophages along with other neutrophils, into the medial layer. Moreover, inflammation and oxidative stress of the arterial wall were induced by endothelial dysfunction. Interestingly, there is a paradoxical differential correlation between diabetes and aneurysm formation in retinal capillaries and the aorta. Deciphering the significance of such a difference may explain current unsuccessful AAA medications and offer a solution to this treatment challenge. It is now believed that AAA and atherosclerosis are two separate but related diseases, based on their different clinical patterns which have further complicated the puzzle. Therefore, a thorough investigation of the interaction between endothelium and medial/adventitial layer may provide us a better understanding and new perspective on AAA formation, especially after taking into account the importance of endothelium in the development of AAA. Moreover, a novel medication strategy replacing the currently used, but suboptimal treatments for AAA, could be informed with this analysis.

## 1. Introduction

By definition, an aneurysm is the dilation of an artery to a diameter at least 50% greater than its normal size. In general, aortic aneurysms are anatomically divided into thoracic aortic aneurysm (TAA) and abdominal aortic aneurysm (AAA) including suprarenal and infrarenal aortic aneurysms. The incidence of TAA each year ranged from 6 per 100 000 in a British study [1] to 9.1/16.3 (F/M) per 100 000 in Sweden and 17.39/21.75 (F/M) in Spain [2, 3]. It is estimated that three quarters of all aneurysms occur in the abdominal aorta. The annual incidence of abdominal aortic aneurysm is 40 for every 100 000 population [4]. Six times more men than women are affected [4, 5]. In elderly males, general infrarenal abdominal aortic diameter is about 15–24 mm. If the diameter exceeds 30 mm, usually more than two standard deviations above the mean diameter for both men and women, this condition is conventionally regarded as AAA [6–8]. A UK survey showed 1.5% of the population had an AAA measuring >30 mm [9]. The Multicenter Aneurysm Screening Study in the USA reported that 4.6% of the population between the age of 65 and 74 years had an AAA [10]. The prevalence of men over 65 years with AAA in Sweden was 1.8% [11]. A meta-analysis of a collection of international studies showed that AAA in men and women is about 6% and 1.6%, respectively [12]. It is suggested that AAA is more common in the clinic than TAA. In recent years, incidence of AAA is trending upward with the aging of the population. Moreover, compared to TAA, AAA has a higher morbidity and poses a greater danger to the population because of its faster rate of growth [8].

Aneurysm rupture is the most serious consequence of AAA. Often the presence of AAA is unknown until rupture, which rapidly progresses to exsanguination and death. The total mortality rate for AAA rupture reaches 90%; therefore, the ideal management is to repair the aneurysm before rupture occurs. However, many aneurysms do not reach surgical indications when they are first discovered, but their size will increase year by year. Blood vessel diameter is strongly correlated with higher rupture risk. It is believed that AAA over 6 cm has a 26% annual risk of rupture [13]. Consequently, AAA has been referred to as “a silently ticking time bomb” in the body [14].

## 2. Anatomy of Abdominal Aorta Is Different from the Thoracic Aorta

Thoracic and abdominal aortas have structural differences in the number of lamellar units, which range from 55–60 U and 28–32 U, respectively [15]. Cells from different segments of aorta have a clear difference in their origin including from the neural crest, mesenchyme, and splanchnic mesoderm, which correspond to different segments. Moreover, the neural crest cell precursors of the thoracic aorta respond differently to various cytokines and growth factors than the mesodermal precursors of the abdominal aorta [16]. Regional differences between the thoracic and abdominal aortas lead to different cellular responses to the same stimuli. For example, the abdominal aorta is susceptible to atherogenesis in contrast to its thoracic counterpart [17]. Moreover, it has been demonstrated that mutations in the transforming growth factor-*β* (TGF-*β*) receptor may lead to TAA but have little effect on the abdominal aorta [18]. This may be explained by the differential function of inflammatory pathways or TGF-*β* between vascular sites. Notably, the medial layer in abdominal aortas normally is completely avascular, whereas the medial layer in thoracic aorta contains vasa vasorum [15]. Therefore, the medial layer of abdominal aorta is more prone to hypoxia than thoracic aorta. Hypoxia is observed in intima and media of AAA lesion because intraluminal thrombus in vascular lesions may prevent luminal perfusion of oxygen, contributing to hypoxia in intima [19]. Furthermore, arteriosclerosis and intimal hyperplasia were shown to induce stenosis of the adventitial vasa vasorum that aggravates tissue hypoxia in the abdominal aorta [20].

## 3. Pathological Mechanisms of AAA

**Figure 1:**
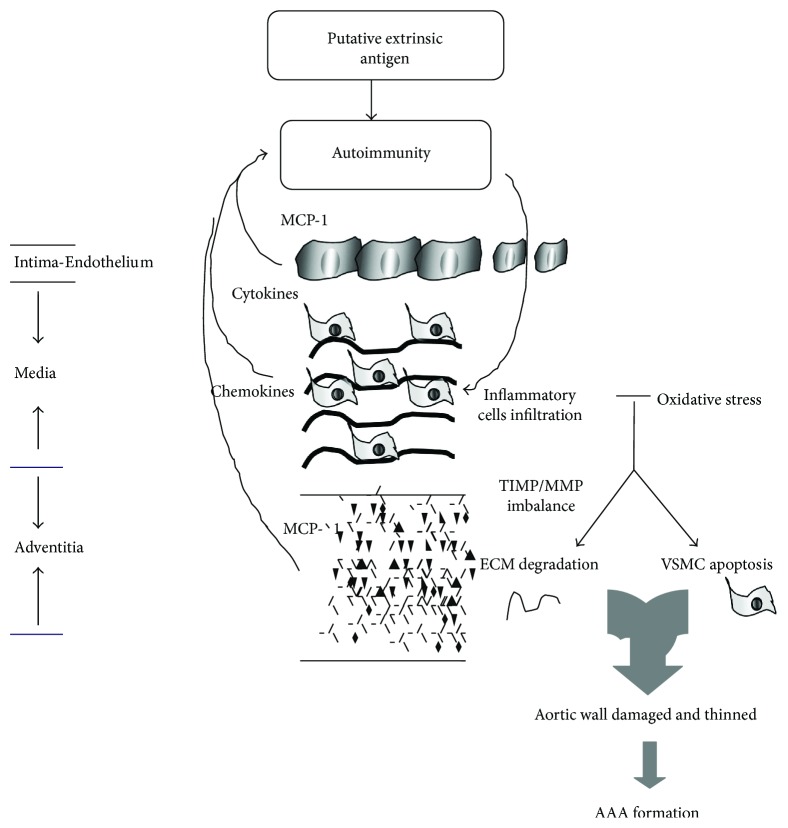
The classic putative mechanism for AAA formation. Extrinsic antigens such as protein from microorganisms cause autoimmunity which can cross-react the medial layer of the aortic wall. This induces and amplifies the autoimmune reaction through inflammatory cell infiltration, cytokine and chemokine production in the three layers of the vascular wall, oxidative stress, and so on. All of these responses further damage the vascular wall via induction of SMC apoptosis and ECM degradation due to TIMP/MMP disorder. VSMCs: vascular smooth muscle cells; ECM: extracellular matrix; TIMP: tissue inhibitors of metalloproteinases; MMP: matrix metalloproteinase; MCP-1: monocyte chemoattractant protein-1.

Although abdominal aortic aneurysmal dilatation is caused by various etiologies leading to complex pathogenesis, histopathological results are always similar; for example, there are degenerations mainly in media and adventitia of aortic wall. Classically, occurrence of aortic aneurysm is associated with aortic wall defects and damage consequent to inflammation, oxidative stress, matrix metalloproteinases (MMPs) activation, and apoptosis of vascular smooth muscle cells (VSMCs). Specifically, inflammatory cells infiltrate in the media and adventitia due to autoimmune reaction with extrinsic antigens, inducing oxidative stress and overproduction of cytokines/chemokines and proteases. This process leads to the breakdown of elastic fibers, degradation of collagen fibers, and loss of VSMCs (Figure 1). As a result, the aortic wall is weakened because of decreased thickness and reduced mechanical function. Eventually, the aortic wall cannot tolerate the impact of blood flow and dilates to form AAA.

## 4. Genetics and AAA

Abdominal aortic dilatation is the outcome of both environmental and genetic factors. In minors, thoracic aortic aneurysm in particular is always the direct consequence of mutations in key genes such as *FBN1*, *COL3A1*, *ACTA2*, and *TGFBR1/2* that causes vassal wall structural defects [21]. However, previous studies have proposed that AAA is a multifactorial disease which involves many genes and pathways, such as extracellular matrix (ECM), inflammation, immunity, oxidative stress, cell signaling, cell growth, and cell survival. Among them, genetics still poses a pivotal role in the AAA development and formation since the risks are higher in those patients with AAA family history than without [22]. Recently, exome sequencing has been carried out to explore possible candidate gene variants by comparing DNA tissue samples from AAA patients and normal controls, in which mutations in 25 genes were found to be associated with AAA formation. However, association studies cannot validate the causative factors for AAA which are still poorly understood [22]. Among them, a missense mutation in ESRRA (estrogen-related receptor alpha), a vascular endothelial growth factor (VEGF) regulator, has been identified [23]. Therefore, there is genetic evidence for endothelium involvement in AAA formation.

## 5. Atherosclerosis and AAA

The infrarenal abdominal aorta is related with the atherosclerotic development and is coincidently the most common site of abdominal aneurysm formation. Previously, it was believed that atherosclerosis is the main pathological process leading to AAA formation. This belief was based on the findings that patients with AAA frequently have concurrent atherosclerosis [24]. However, it has been suggested that aneurysm formation and atherosclerosis do not develop in parallel, but through different pathogenic mechanisms independently [24]. For example, the lumen narrows in atherosclerosis but grows larger in AAA; diabetes precipitates atherosclerosis but protects against AAA; and proliferation of SMC happens in atherosclerosis but apoptosis of SMC in AAA. Therefore, AAA and atherosclerosis are now regarded as two separate but related diseases. Indeed, atherosclerosis and AAA share some common risk factors such as both are closely associated with endothelial dysfunction [24].

## 6. Animal Evidence for Endothelial Dysfunction in AAA

It is generally believed that the receptor inducing cell response to increase tension in the aortic wall produced by Ang II originates in the medial layer but not in the endothelium. Conversely, endothelial cell-specific deficiency of Ang II type 1a receptors markedly attenuates the development of Ang II-induced aortic aneurysms in LDL receptor^−/−^ mice, but Ang II type 1a receptor deficiency in VSMCs had no such effect on the development of aneurysms [25]. Moreover, Franck et al. demonstrated that restoring the endothelial lining is an efficient therapy to control AAA dynamics and stop AAA expansion [26].

Gradually, the endothelium drew the attention of AAA research. Endothelial cells are single layer of squamous epithelium lining the vessel lumen and participate in many physiological activities to maintain the normal structure and function of the vessel wall. In the arterial wall, endothelial cells secrete a variety of substances that influence the function of other cells, including smooth muscle cells, white blood cells, and so on, to initiate a critical series attacks on media and adventitial layers. The pathological changes of endothelial cells or endothelial dysfunction are possible earlier than those of media and adventitia in the process of AAA formation.

Deficiency of eNOS increases atherosclerosis and AAA in *apoE* (−/−) mice without altering arterial blood pressure, body weight, serum cholesterol concentrations, or distribution of lipoprotein cholesterol [27, 28]. Moreover, Ang II-infused apoE (−/−) or PCSK9 overexpression mice is induced by hypercholesterolemia and AAA [29]. The endothelial function is regulated by NO bioavailability whose production is modulated by eNOS expression and coupling status. However, eNOS uncoupling leads to endothelial dysfunction, promoting excessive oxidative stress in endothelial cells which induces harmful oxidative/nitrosative stress to local cells.

The coupling status of eNOS is determined by its cofactor tetrahydrobiopterin (HB_4_), whose production is governed by a de novo synthetic pathway (the key enzyme being GTP cyclohydroxylase 1 (GCH1)) and a salvage pathway (the key enzyme being dihydrofolate reductase (DHFR)). It has been reported that the mouse model (hph1) with HB_4_ deficiency due to GCH1 mutation was characterized as having eNOS uncoupling and endothelial dysfunction, which led to a high risk of AAA with Ang II infusion [30]. Recoupling eNOS with HB_4_ supplementation or folic acid treatment prevented AAA formation or reduced such risk [30]. Moreover, H4B levels in serum and the aorta are closely correlated in which both decreased with AAA development but increased with folic acid treatment in Ang II-infused hph1 or apoE (−/−) mice, suggesting serum H4B can be used as a biomarker for AAA development and treatment assessment [31]. Furthermore, Cai et al. reported that animals possessing the double mutation of hph1 and NOX1, NOX2, p47phox, or NOX4 had reduced AAA incidence and abdominal aortic expansion after Ang II infusion due to reduced superoxide production, as well as improved NO and H4B bioavailability, and restored eNOS coupling activity compared to hph1 mice. These changes preserved DHFR function in the endothelium [32].

## 7. Clinical Evidence for Endothelium Involvement in AAA

A clinical study demonstrated that circulating biomarkers of endothelial dysfunction in atherosclerosis patients (white blood cell count, fibrinogen, D-dimer, troponin T, N-terminal pro-brain natriuretic peptide, and high-sensitivity C-reactive protein) are also closely related to the occurrence and incidence of AAA [33]. Epidemiological surveys show that AAA risk factors, such as aging, smoking, male sex, high blood pressure, hyperlipidemia, atherosclerosis, and hyperhomocysteinemia, are the main factors that cause damage to endothelial cells leading to endothelial dysfunction [34–36]. For instance, the prevalence of AAA in the population who smoke is four times greater than those who do not smoke, meaning smoking is detrimental to endothelial function and thus closely related to many cardiovascular diseases [34, 37]. Furthermore, recent studies in humans indicate that some endothelial protective medications such as statins, angiotensin-converting enzyme inhibitors (ACEIs), and AT_1_ receptor blockers (ARBs) may be helpful to inhibit aneurysm rupture and growth, yet the effect is unclear in some larger cohort reports [38, 39]. Mechanism of medication is also not clear, so the effect of regulating endothelial function and increases in nitrogen oxide (NO) cannot be ruled out.

## 8. Endothelium and Shear Stress

The endothelium is in direct contact with blood flow which can produce a large amount of shear stress. The influence of different flow patterns on vessel walls produces two kinds of shear stress: unidirectional laminar shear stress (LSS) and oscillatory shear stress (OSS). It has been reported that AAA occurs in areas of reflux, low wall shear stress, or blood flow disorders, while high LSS reduces the development of AAA [40]. Hemodynamic forces regulate oxidative stress and reactive oxygen species (ROS) in the endothelium. LSS inhibits superoxide while OSS increases that from endothelial cells. Additionally, NOX mediates oxidative stress in response to distinct shear stresses [41]. LSS activates NOX2 to induce NO production, while OSS activates NOX1 with uncoupled eNOS [41]. Furthermore, shear stress decreases the expression of eNOS in endothelial cells via a receptor named scavenger receptor class B member-1 (SR-B1) [42]. SR-B1, a receptor for high-density lipoprotein, is involved in the regulation of endothelial cells in response to shear stress, which contributes to AAA formation in some patients with atherosclerosis and some animal models such as Ang II-infused ApoE gene knockout mice [28].

Shear stress regulates the expression and activity of proteases secreted by endothelial cells. Normally, ECM is composed of elastin, collagen, proteoglycan, glycoprotein, glycosaminoglycan, and so on. ECM maintains an equilibrium between synthesis and degradation, while patients with AAA have disorders in ECM metabolism, manifesting as lesions of elastin and collagen fibers [43]. MMPs and cysteine proteases, such as cathepsins K, L, and S, participate in the degradation of ECM [44, 45]. Accordingly, expression of TIMP3 decreases and MMPs activity increases in relation to the condition of OSS compared to LSS [46]. Although some studies show that the TIMP is unexpectedly increased in the wall of the aneurysm [47], the balance between proteases and antiproteases seems to favor proteolysis [7]. Cathepsin is another kind of protease family that hydrolyzes the ECM, destroying the elastic and collagen fibers to contribute to the formation of AAA [45]. Expression of cathepsin is observed to be increased in endothelial cells at the site of AAA lesions [48, 49]. The expression and activity of cathepsins are significantly lower under laminar flow than those under turbulent flow in the endothelial cells [50, 51]. Hemodynamic forces indirectly mediate the expression of cathepsin in endothelial cells by regulating infiltration of inflammatory cells and release inflammatory factors [51, 52].

## 9. Endothelium and Inflammation and Oxidative Stress

Endothelial cells are involved in the inflammation of the aortic wall. Shear stress and blood flow on the endothelium influence the aortic wall via inhibition of inflammatory cytokines and adhesion molecules secreted by endothelium, further reducing the inflammatory reaction in the media and adventitia [53]. Based on previous studies, it is possible that AAA formation is triggered by innate or autoimmunity to extrinsic antigens that may share molecular motifs with those on the aortic wall [54]. Inflammation has been considered to be an essential factor in the initiation and progression of aneurysms. Infiltrating macrophages and leucocytes are major sources of proteinases. Furthermore, infiltrating immune cells release cytokines, ROS, and cellular adhesion molecules, which lead to further recruitment of immune cells, induction of VSMC apoptosis, and tissue injury.

Evidence shows that transcription factor nuclear factor-kappa B (NF-*κ*B) mediates a number of genes associated with both inflammatory and oxidative reactions in the aortic wall [55–57]. It was found that when the NF-*κ*B pathway of endothelial cells was blocked, infiltration of inflammatory cells, expression of inflammatory factors, and oxidative stress response were reduced in the media and adventitia of arteries; this led to the inhibition of aneurysm formation [58]. Hannawa et al. found that selectins promote the recruitment and infiltration of inflammatory cells in early AAA formation [59]. They further demonstrated that inflammatory cell recruitment was significantly diminished in P-selectin knockout mice, so deficiency of P-selectin, which is expressed in endothelial cells, attenuated aneurysm formation [60].

Endothelial cells are also involved in oxidative stress of the aortic wall. Endothelial cells are stimulated by secreted substances that increase the level of ROS in smooth muscle. The occurrence of oxidative stress in endothelial cells is observed before the changes in VSMCs as well as infiltration of inflammatory cells. Oxidative stress and inflammation interact to increase damage to arterial tissues. At the site of tissues with chronic inflammation, increased level of ROS is generally observed. Inflammatory mediators were shown to activate NADPH oxidase to produce O_2_^−^, and NADPH oxidase as well as iNOS, in turn, participates in inflammation reaction [61, 62]. ROS products promote the infiltration of inflammatory cells and increase the secretion of proinflammatory cytokines [63]. Besides these roles, ROS directly activates MMPs [64], inhibiting plasminogen activator inhibitor type-1 (PAI-1) which is a MMP inhibitor that induces apoptosis of VSMC [65]. This increase in ROS in endothelial cells altered the function of VSMCs and promoted oxidative stress, in which cyclophilin A (CypA) may act as an intermediate molecule [66]. CypA induces smooth muscle cell migration and proliferation, increases the expression of endothelial adhesion molecules, and mediates the chemotaxis of inflammatory cells [66]. After specific knockdown of the CypA gene in endothelial cells, however, Ang II did not increase the ROS level in VSMCs, in which secretions of inflammatory cytokines such as MCP-1, IL-6, and chemokine were also blocked [67]. Moreover, levels of CypA in VSMCs are also influenced by endothelial cells [66].

## 10. ECM and VSMCs in Media

ECM and VSMCs are chief components in media tissue. On the one hand, VSMCs decrease in number because of apoptosis and necroptosis [68], and on the another hand, VSMCs synthesize large amounts of ECM components and increase proliferation as well as migration in the vascular wall. This is one of the most significant reasons for media tissue weakening that leads to structural and functional abnormalities in apoptotic VSMCs [69]. Wang et al. reported that macrophage recruitment caused VSMCs death through a FasL/Fas-Caspase-8-RIP1-mediated pathway [70]. RIP1 inhibitors reverse VSMC loss, prevent inflammation and ECM degradation, and promote aortic tissue repair. Additionally, VSMCs are essential not only for short-term regulation of blood pressure via alteration of vessel tone and diameter, but also for long-term adaptation via structural remodeling by changing cell number and connective tissue composition [71]. As contractile properties decrease, proliferation and migration increase in VSMCs of AAA lesions as result of transforming to a synthetic phenotype. It has been demonstrated that inhibition of the mTOR cascade attenuates AAA progression by preserving or restoring VSMC contractile phenotype [72].

Compared to nonruptured aneurysms, angiogenesis is more obvious in the external two thirds of the media tissue in ruptured AAA. The infiltration of inflammatory cells and inflammatory factors causes medial lymphangiogenesis and angiogenesis [19]. These neovessels are incomplete, leaky, and rupture easily, resulting in vascular remodeling and weakening the aortic wall by weakening its structural integrity [73, 74]. Moreover, formation of lymphatic microvessels may introduce inflammatory cells into media tissue and newly formed lymphatic microvessels cannot drain lymph fully [19]. However, the exact mechanism of neovascularization and lymphangiogenesis in aortic aneurysm rupture and development is not clear.

## 11. Adventitia and AAA

Both the thoracic aorta and the abdominal aorta are elastic arteries that consist of an intimal, medial, and adventitial layer. The alteration of elastin and collagen in the aortic wall is dependent on production of proteases by resident vascular wall cells (medial smooth muscle cells and adventitial fibroblasts) and lymphomonocytic infiltrate cells, a process in which adventitia plays a major role. Notably, adventitial fibroblasts secreting MCP-1 have also been shown to recruit monocytes to the aortic wall that then promote the proliferation of more fibroblasts to amplify the inflammatory response. This amplification represents the pivotal step in AAA development and formation [75].

## 12. Signaling Pathway and AAA

**Figure 2:**
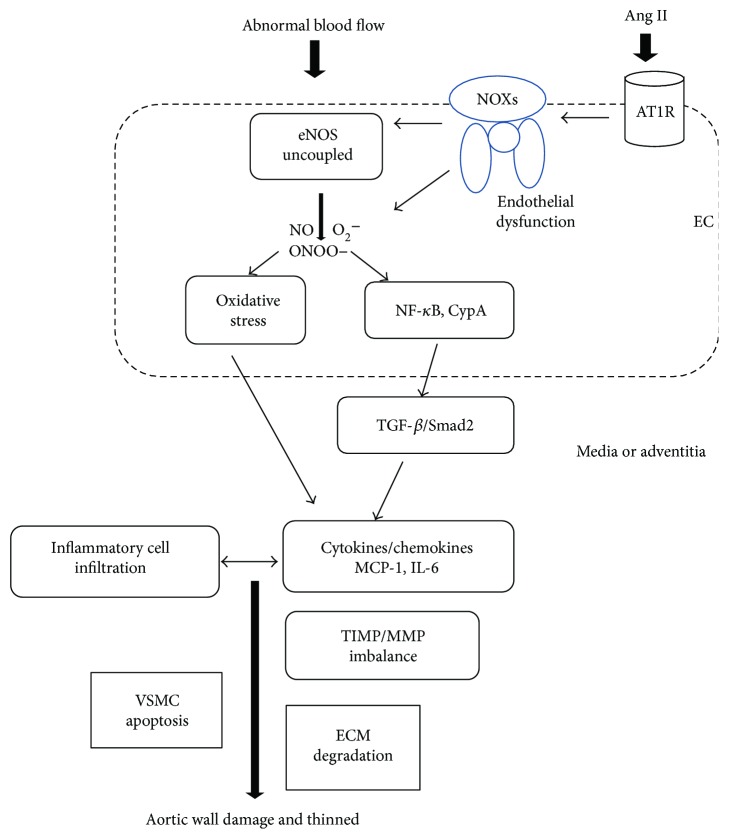
The proposed role of the endothelium in AAA formation. Shear stress produced by blood flow together with Ang II influences the endothelium to cause endothelial dysfunction via uncoupling of eNOS and oxidative stress induction. Endothelial dysfunction activates NOXs, NF-*κ*B, CypA, and TGF-*β*/Smad2 pathways to further initiate and amplify the inflammatory reaction via overproduction of cytokines and chemokines such as MCP-1 and IL-6. More inflammatory cells infiltrate and exacerbate oxidative stress to form a vicious cycle. The balance of TIMP/MMP is tipped, and proteolysis is promoted. Eventually, the aortic wall is damaged as a result of SMCs apoptosis and ECM degradation due to proteolytic degradation. EC: endothelial cell; Ang II: angiotensin II; AT1R: AT_1_ receptor; eNOS: endothelial nitric oxide synthase; NO: nitric oxide; O_2_^−^: superoxide anion; ONOO^−^: nitrous oxide ion; NF-*κ*B: nuclear factor-kappa B; CypA: cyclophilin A; TGF-*β*: transforming growth factor-*β*; MCP-1: monocyte chemoattractant protein-1; IL-6: interleukin-6; TIMP: tissue inhibitors of metalloproteinases; MMP: matrix metalloproteinase; ECM: extracellular matrix; VSMCs: vascular smooth muscle cells.

Many mechanisms have been proposed for AAA formation, and basic research has helped to determine the molecular basis and mediators of aortic damage including angiotensin II, leukotriene-LT4, prostaglandin E2 (PGE2), interleukins, tumor necrosis factor, tissue plasminogen activator, c-Jun N-terminal kinase, NF-*κ*B, rho kinases, osteoprotegerin, chymases, hypoxia-inducible factor-1 (HIF-1), metabolism, SMAD, TGF-*β*, and its signaling pathway [23, 76, 77]. The overall mechanism is still a puzzle, but the current knowledge about AAA formation is illustrated in Figure 2: in the presence of some aggravating factors such as hypertension, smoking, and aging, NOXs are activated upon RAS activation which further causes eNOS uncoupling via oxidation of H4B (cofactor for eNOS) or oxidative stress/superoxide overproduction. Endothelial dysfunction is consequential to eNOS uncoupling, which triggers a series of cascades including overexpression of ICAM/VCAM to amplify the immune response and induce monocyte infiltration. These mechanisms expand the oxidative stress to media and adventitia layers. Taken together, MMP activity is induced to initiate ECM degradation and proteolysis while VSMC becomes apoptotic to further weaken the vascular wall. NF-*κ*B is also activated, and collagen lysis cannot be balanced by fibroblast. Moreover, the intraluminal thrombosis in adventitia-induced hypoxia causes neovascularization.

## 13. Diabetes and Aneurysm in Retina Capillary versus Aorta

Retinal capillary aneurysm is a marker of very early diabetic retinopathy (DR), in which diabetes is regarded as a very high risk factor for retinal capillary microaneurysm [78]. Paradoxically, epidemiological data for AAA showed a negative association with diabetes [79–82], although some conflicting data exist [83, 84]. The prevalence of type 2 diabetes in patients with AAA ranged from 6 to 14%, while it was as high as 17 to 36% in the absence of AAAs [85]. It was also found that the growth rate of aneurysms in patients with diabetes is on average lower than in nondiabetics [86, 87]. The mechanism is not yet clearly known. However, despite the negative association, there is a higher mortality rate in diabetes with AAA than nondiabetic patients with AAA [85].

Pericytes covering capillaries have functions similar to smooth muscle cells, that is, maintaining the mechanical stability of capillary structure. Retinal microaneurysm is an early pathological change in diabetic retinopathy, characterized by the disappearance of pericytes from retinal capillaries. The role of endothelium in DR is not clear. However, recently, it was discovered that hyperglycemia induced PKC activation which had inhibitory effect on eNOS expression in endothelial cells inducing endothelial dysfunction [88]. Moreover, it has been reported that hyperglycemia promotes leukocyte adhesion to the endothelium possibly through upregulation of NF-*κ*B activation which causes apoptosis of endothelial cells [89, 90]. Advanced glycated end products (AGEs) induced by hyperglycemia are bound to its receptors in endothelial cells and pericytes leading to retinal microvessel damage by influencing intracellular functions, such as increased ROS production, vascular stiffness, and cell apoptosis. On one hand, apoptosis of endothelial cells and pericytes in situ position, while futile endothelial cell proliferation in transposing position without pericyte support, both of which lead to microaneurysm formation in the retina.

AAA mice with hyperglycemia demonstrated diminished macrophage infiltration, elastolysis, and neovascularization in their aortic walls [79]. Research has shown that AGE accumulation is negatively correlated with aortic diameter [91] and MMP-2 expression and activity [92]. It is also postulated that medical treatment for diabetes, like metformin, may have a protective effect on AAA. Nevertheless, it seems that retinal microaneurysm formation is different from aortic aneurysms in diabetes. A thorough investigation of the differential correlation between diabetes and aneurysm formation in retinal capillary versus aorta may shed light on a current gap of AAA research.

## 14. Current Treatments for AAA

At present, surgical repair of expanding arteries or stent installation is the primary measure used to reduce the risk of aneurysm rupture. Whether patients require surgical treatment is dependent on a comprehensive evaluation of many factors, such as the diameter of aneurysm, its growth rate, the presence of symptoms, and life expectancy. Common clinical surgical methods include open surgical repair and endovascular aneurysm repair (EVAR). In EVAR, a tube or stent is implanted into the abdominal aorta cavity to uphold the aortic wall and allow blood flow to pass through the vein. Although, compared with conventional open surgical repair, patients given with EVAR have a short postoperative recovery time and low short-term mortality, there is also no significant difference in long-term (>2 years) mortality.

Patients with a symptomless aneurysm diameter of <40 mm with a low risk of rupture are recommended to be treated with conservative treatments and receive regular reexamination. It is still controversial as to whether patients with an aneurysm size of 40–54 mm should undergo surgery. Some studies have shown that regular color Doppler ultrasound or abdominal aorta CTA examination to monitor the growth of the aneurysm is an effective and safety method. There was no significant improvement to long-term survival rate when these patients underwent early surgical treatment. Therefore, surgical indications of patients with an aneurysm diameter of <54 mm greatly depended upon individual circumstances. The elderly with severe complications, especially, is recommended to receive conservative treatments. Screening of high-risk populations, such as the elderly, smokers, and people with a relevant family history, to detect biomarkers in blood is beneficial for early diagnosis and treatment to reduce the mortality from ruptured aneurysms [93, 94]. It is important that patients who do not have surgical indications should still receive medical treatment in order to limit aneurysm growth.

While surgery is the only measure used to reduce the risk of aneurysm rupture currently, noninvasive medical treatments can be used as adjuvant therapies. A variety of anti-inflammatory, antioxidant, and hemodynamic modulator drugs and MMP inhibitors are currently being studied in order to slow aneurysm growth, reduce the risk of rupture, and improve prognosis as well as mortality after surgery. The growth rate of aneurysm growth is increased in people who smoke, so lifestyle interventions to quit smoking are beneficial in preventing AAA occurrence and delay the aneurysm development [87].

Statins (3-hydroxy-3-methylglutaryl coenzyme A reductase inhibitors) reduce the level of ROS products and have anti-inflammatory effects to limit the progress of AAA independent of lipid-lowering effects [95, 96]. Renin–angiotensin–aldosterone system inhibitors, ACEI and ARB, should theoretically slow the arterial dilation and reduce the risk of AAA rupture. While statins and ACEI are well tolerated, a recent meta-analysis on the available data concluded that these drug classes or anti-inflammatory therapies did not influence AAA progression [97]. Although animal studies have demonstrated that beta receptor blockers can inhibit the growth and rupture of aneurysms by influencing hemodynamics, which is beneficial to delay AAA expansion [98], there was no clinical evidence for a beneficial effect of such strategies on AAA progression. On the contrary, evidence was found for growth acceleration in patients taking doxycycline, a MMP inhibitor [99]. This evidence contrasts sharply with the available preclinical data that shows the pharmaceutical interference in aspects of the RAS system, cholesterol metabolism, vascular inflammation, or protease activity effectively alleviates aneurysm formation in rodent disease models [100]. Consequently, although cardiovascular risk management does not influence AAA progression, it is important to realize that risk management is indicated in AAA patients as this group is at an extremely high cardiovascular risk [6].

A pharmacological approach has not been identified that effectively limits AAA progression or the risk of rupture in humans. What has been lacking is a detailed understanding of the mechanisms of AAA initiation and expansion. Studies on the role and significance of vascular endothelial cells in AAA pathogenesis and progression will give a new perspective for a novel target discovery for the prevention of AAA formation and delaying its progression. Statins, anti-RAS drugs, and antioxidants may have some effects on the restoration of endothelial dysfunction but are not effective or specific in eNOS recoupling [30, 101–105]. Folic acid or other drugs by targeting eNOS uncoupling should be developed and trialed in AAA to test their effectiveness [30, 106].

## 15. Conclusion and Perspective

AAA is a potentially fatal cardiovascular disease, and it will become more and more common as the population ages. The pathological mechanisms of AAA are the result of many factors, including a decrease in VSMCs, MMPs activation, breakdown of ECM, and inflammatory cell infiltration, but the role of endothelial cells cannot be ignored. With the recognition of the importance of endothelial dysfunction in AAA formation as well as validation of the effectiveness of pharmacological therapies in AAA, we may discover a promising strategy for early intervention for high-risk patients or a surgical adjuvant therapy to complement current surgical options in advanced AAA. To this end, the differential role of endothelial function on the development of capillary microaneurysm and AAA in diabetes may underlie a promising solution for AAA.
